# Therapeutics

**Published:** 1877-12

**Authors:** 


### II. Therapeutics.

Effects of Sulphate of Atropia on the Nervous System.—Sidney Ringer. (*Journal of Anatomy and Physiology*, *from Edinburgh Medical Journal*, July, 1877.) From his experiments, Ringer concludes that the late occurrence of tetanus in atropia poisoning, is not due to paralysis of the motor nerves, but that it is owing to the cord being slowly affected. It appears that whilst the poison very quickly paralyzes, it takes many hours, or even days, before it tetanizes. He also concludes that atropia paralyzes much more through its depressing action on the spinal cord than on the motor nerves, and that it has a direct paralyzing action on the cord, and does not affect it through its depressing action on the circulation. In atropia we have a drug which quickly paralyzes the reflex functions of the cord, but requires a much longer time to diminish the resistive power of the cord; hence, paralysis precedes, and may even disappear, some hours before the onset of tetanus.

Bromide of Arsenic in the Treatment of Nervous Diseases.—Clemens. (*Allg. Med. Central-Zeitung.*) Cl. states that he has obtained astonishing results with bromide of arsenic in the treatment of diseases of the nervous system, and especially epilepsy. The following is the formula which he thinks should replace Fowler's solution: Pulv. arsenic alb., potass. carb., aa ʒj.; coque cum aq. dest., ℔. ss. ad solut. perfect.; aq. evaporat. adde aq. dest., ℥xij.; brom. pur. ʒij. refrigerat. S. arsenic bromatum. Of this he gives one or two drops in a glass of water once or twice daily. This dosage may be continued for months, or even years, without producing any unpleasant effects. In only two cases of epilepsy did he effect a complete cure, but in all the cases, marked relief was obtained. In connection with the bromide of arsenic an almost exclusively meat diet is advised. The patients should be as much as possible in the open air. Unlike the bromide of potassium, the arsenical salt does not require to be given in increasing doses, and, instead of interfering with digestion, improves the nutrition and strength.

Action of the Salts of Silver on the Muscular and Nervous Systems.—Curci. (Giornal. Veneto di Scienze, from *Edinburgh Med. Journal*, July, 1877.)

In a series of experiments on the action of silver on the muscular and nervous systems, Curci used one part of chloride of silver, three parts of hypophosphite of soda, and thirty of distilled water. Administered thus, silver does not irritate the skin or mucous membrane. Injected subcutaneously, it produces slight inflammation and œdematous swelling, but is easily absorbed, and does not coagulate the blood. Curci concludes that it acts on the sensory nerves, and through them on the posterior column of the cord. It first stimulates them and increases sensibility to pain, raises reflex excitability, and extends its action to the motor portion of the cord, producing tetanus and increased muscular tone. It increases muscular irritability, and paralyzes secondarily the sensory nerve-centres, especially the respiratory centre. At length it annihilates reflex excitability, respiration and circulation cease, and the heart remains in a state of diastole. C. thinks that these results show the inefficacy of silver in the diseases for which it has been hitherto in repute—myelitis, paralysis agitans, and locomotor ataxy. Where there is softening or induration, proliferation of connective tissue, and destruction of nerve-elements, and where the muscular tone is weakened, no good action can be expected from a medicine which itself produces these conditions. Silver may be used beneficially in cases of epilepsy which depend on excessive irritation of the spinal cord, but it exerts no effect in those due to anatomical lesions. In hysteria it is useless, but is beneficial in chorea. Its administration in nervous asthma may be attended with excellent results, and as it reduces the irritability of the respiratory centre, it would most probably be especially useful in cases attended with spasm of the inspiratory and bronchial muscles.

